# From Attitudes to Actions: Predictors of Lion Killing by Maasai Warriors

**DOI:** 10.1371/journal.pone.0170796

**Published:** 2017-01-30

**Authors:** Leela Hazzah, Alistair Bath, Stephanie Dolrenry, Amy Dickman, Laurence Frank

**Affiliations:** 1 Nelson Institute for Environmental Studies, University of Wisconsin, Madison, United States of America; 2 Living with Lions, Nanyuki, Kenya; 3 Memorial University, Human Dimensions in Wildlife Management, Department of Geography, St. John’s, Newfoundland and Labrador, Canada; 4 Wildlife Conservation Research Unit, Department of Zoology, University of Oxford, The Recanati-Kaplan Centre, Tubney House, Tubney, Oxford, United Kingdom; 5 Museum of Vertebrate Zoology, University of California Berkeley, United States of America; Panthera, UNITED STATES

## Abstract

Despite legal protection, deliberate killing by local people is one of the major threats to the conservation of lions and other large carnivores in Africa. Addressing this problem poses particular challenges, mainly because it is difficult to uncover illicit behavior. This article examined two groups of Maasai warriors: individuals who have killed African lions (*Panthera leo*) and those who have not. We conducted interviews to explore the relationship between attitudes, intentions and known lion killing behavior. Factor analysis and logistic regression revealed that lion killing was mainly determined by: (a) general attitudes toward lions, (b) engagement in traditional customs, (c) lion killing intentions to defend property, and (d) socio-cultural killing intentions. Our results indicated that general attitudes toward lions were the strongest predictor of lion killing behavior. Influencing attitudes to encourage pro-conservation behavior may help reduce killing.

## Introduction

The conservation of large carnivores poses substantial challenges [1, 2] due to these species’ wide-ranging nature and high potential for livestock depredation [3, 4]. Killing by humans has eliminated many carnivore species from extensive portions of their former range, including puma (*Puma concolor)*, tiger (*Panthera tigris)* and African lion (*Panthera leo*) [3, 5, 6]. As human and livestock populations have rapidly expanded, large carnivore habitat and prey have declined and human-carnivore conflict over depredation has increased [6, 7]. Carnivore killing may be motivated by the degree of damage (e.g., livestock depredation) incurred [8, 9], but social and cultural beliefs are often equally important drivers and need to be addressed in order to reduce killing [10, 11].

African lions have declined by at least 43% over the past 21 years [12] and the IUCN estimates that between 20,000 and 39,000 remain on the African continent, with the figure probably closer to 20,000 [13]. East Africa supports nearly 60% of the remaining lion population [14] and indiscriminate killing poses the greatest threat in this region [13]. Many lions are illegally speared, shot, and poisoned annually across East Africa [15–17]. High levels of killing, even at a local level, can have far-reaching impacts on lion populations [17–19].

Understanding the factors that trigger lion killing could provide insight into potential solutions. Research has shown that people living with lions often hold negative attitudes toward them based on negative experiences (e.g., livestock depredation) [16, 20, 21]; however, direct benefits or perception that lions are culturally important can improve attitudes [22–24]. Albarracín, et al. [25] define attitude as a physiological tendency of an individual to evaluate an entity (person, place, behavior or thing) with a degree of favor. There are, however, many studies that suggest that general attitudes toward a target species/protected area do not always predict behavior (see [26] for full table). Understanding the specific relationship between attitudes and actions toward a species is critical for developing locally-appropriate conservation initiatives.

We examined attitudes toward lions among the Maasai pastoralists in southern Kenya including their engagement of traditional customs, as adhering to traditional customs and faith may reduce the propensity toward wildlife killing [27–29]. Hazzah et al. [20], for example, found that Maasai who abandoned traditional customs and adopted evangelical Christianity reported a higher propensity to kill lions. Likewise, in Tanzania, respondents who adhered to a formal religion rather than traditional belief systems reported higher conflict with lions and other large carnivores [15]. There were similar findings in Indonesia, where the conversion to Christianity lead to an increase in the likelihood of hunting [30]. The stronger the cultural acceptance of domination of wildlife, the more likely attitudes and actions will result in wildlife killing [31, 32].

St. John et al. [26] suggest that understanding motivations and intentions toward wildlife killing could help predict behavior. Considerable research has focused on understanding the motivations and characteristics of licensed hunters, particularly in North America [33, 34], but there is little information regarding those individuals who kill an animal illegally or as part of more traditional lifestyle that follows cultural norms [35, 36]. Lion killing is illegal in Kenya, but openly discussed and celebrated by the Maasai in southern Kenya; with those who kill lions gaining respect within their communities. For example, the first warrior to spear a lion in the course of a hunt is awarded a ‘lion name’ (each warrior gets only one lion name even if he kills multiple lions), which he is known for the rest of his life. This name is one that blends the perceived character of the warrior with that of the lion killed, and provides lifelong prestige to the individual [37–39]. This open context provided an opportunity for examining the characteristics of known killers and their attitudes and intentions toward lion killing.

The severity and complexity of wildlife killing is widely acknowledged [40], but little empirical research has focused on predicting wildlife-killing behavior, often because measuring actual behavior is difficult, particularly when it is illegal [26]. As a result, effort has been focused on methodologies that uncover illicit behavior [41–44]. In this study, however, we had data on known lion killing as we could recognize lion killers through their lion names, as well as on general attitudes toward lions, killing intentions, and Maasai engagement towards traditional customs. Our objective was to identify variables that most strongly predict actual lion killing behavior. McCleery [45] suggests that to truly understand illicit behavior, collecting data on behavioral intentions and attitudes of those with experience (e.g., lion killing) could then be analyzed as a subset and compared to those without the experience. We took this approach and surveyed Maasai who have killed lions and those who have not. Understanding what triggers lion killing in rural settings, like Maasailand, could provide insight in designing more effective and targeted solutions to predict and ultimately reduce illegal wildlife killing.

### Maasai Culture and the Significance of Lion Hunts

Lion killing has traditional significance within the Maasai society. Maasai are organized socially by gender and age-class, which determines social relations and community responsibilities [23, 46]. Warriors are the youngest group of men (approximately 15 to 30 years of age), who value bravery and respect from their peers and the broader community [47]. Their primary responsibility is to defend their community from other warriors stealing their cattle and from predatory wildlife. The relationship between Maasai and lions is multifaceted, and includes positive and negative attitudes [23] and must be evaluated in order to understand lion killing tradition.

Historically, killing a human enemy or a lion were comparable feats in gaining prestige and renown. Today, killing human enemies is rare but killing a lion is still important to a young warrior’s attaining manhood, as he is seen to be protecting his community. Lion hunts are a means of transmitting masculine values between generations and the warriors are the caretakers of this tradition. The Maasai have historically valued lions (except when they have attacked livestock) because they provide warriors with a cultural role that reasserts their power and strength as they protect their communities [48]. Some Maasai have more recently come to value lions for perceived tourism value [49].

We use the term “killers” rather than “hunters” since hunting is defined as “the practice of chasing and killing an animal or bird for food, sport/recreation, or profit:” [50]. The Ilkisonko Maasai refer to lion killers as ‘etaara olowurau’, literally translated to ‘killed a lion’; thus the term “lion killing” is more accurate within this cultural context.

### Study Area

**Fig 1:**
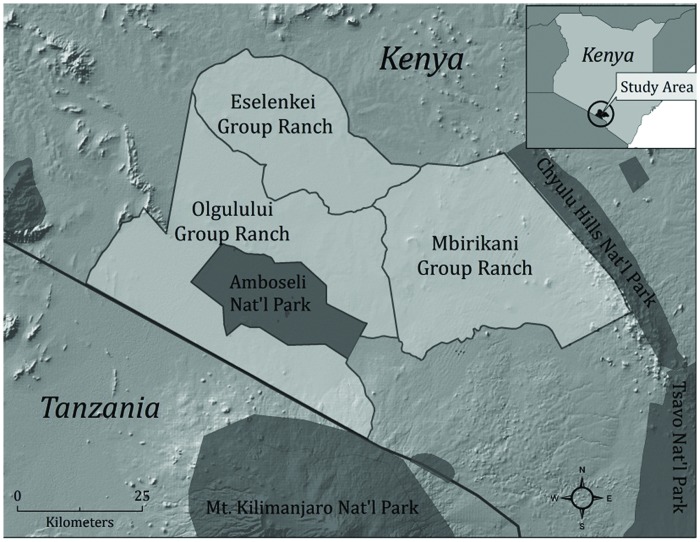
Map of study areas within the Amboseli-Tsavo ecosystem.

We studied three communities in the Amboseli-Tsavo ecosystem in southern Kenya; Mbirikani (1,229 km^2^), Olgulului (1,470 km^2^), and Eselenkei (748 km^2^) Group Ranches (Fig 1). The group ranches are communally-owned land and have similar ethnic, economic, and ecological characteristics. Residents are Maasai pastoralists with a low level of economic prosperity, and pastoral livestock production remains their dominant livelihood activity [51]. These group ranches form part of several corridors that connect wildlife from four national parks (Amboseli, Tsavo, Chyulu Hills, Kilimanjaro) in the greater Amboseli-Tsavo ecosystem [52]. In 2009, approximately 27,000 Maasai lived on the three group ranches, and many experienced livestock depredation by carnivores [53–55]. The lion population of the group ranches was approximately 150 total individuals in 2015 [56]. Lions were killed in response to livestock depredation, in traditional unprovoked hunts by warriors, political protest, and in defense of property and life, with a minimum of 160 lions speared or poisoned by Maasai in the three focal group ranches between 2003 and 2011 [57].

## Materials and Methods

### Ethics Statement

This research was approved under The University of Wisconsin- Madison IRB approval number E-2005-0222. Because most respondents were non-literate, all were read a consent clause and provided verbal rather than written consent before being interviewed. To ensure anonymity, respondents’ identities were immediately codded numerically and no names were collected.

### Sampling and Survey Instruments

A total of 123 semi-structured interviews were conducted in the study area between January and June 2010 (35% on Mbirikani, 23% on Eselenkei, and 42% on Olgulului). We used a criterion sampling framework (i.e., a process of identifying individuals with specific attributes relevant to the study's purpose [58], with individual warriors being our sampling unit. Only Maasai warriors living within the study area were interviewed, as this segment of society was primarily responsible for killing lions. A mixed-methods approach was used to verify the history of all lion killers, who were identified through their lion names. Each alleged hunt was recounted independently by at least three participants who were present at the hunt, and these accounts were compared (‘triangulation’) [57].We interviewed 61 individuals who had killed at least one lion in the past five years, and then selected an additional 62 respondents with similar characteristics to the ‘killers’ (e.g., were physically fit and able to kill a lion, from the same age-set, had little to no formal education, similar livestock holdings, spent the majority of their life herding livestock, and resided in the same communities as the identified lion killers) with no lion killing experience (non-killers), to avoid bias.

The questionnaire was pilot-tested with 25 different respondents between October and December 2009. All questionnaires were written and conducted in the Maa language by a single interviewer to minimize interviewer bias [59]. ‘Translation/back-translation’ was used to increase the reliability of the translated questionnaire [60]. This was done twice with two different translators to ensure accuracy.

Attention was paid to pre-testing the ‘engaging in traditional customs’ scale (henceforth called, “traditional customs”) to ensure optimal reliability and validity. Since culture is dynamic and may vary regionally, it was important to first identify the underlying cultural dimensions necessary to build the scale. Over a two-month period, three focus group discussions were held. During these discussions, Maasai men of all ages developed a list of 100 items that best described Ilkisonko Maasai traditional customs in the Amboseli region. These items were subsequently ranked based on perceived importance by individual elders and warriors [61] and then reduced to the five most frequently mentioned items for the final scale (traditional customs). The attitudes toward lions and killing intentions scales were developed via a literature review [62, 63] and common ethnographic inquiry: prolonged engagement, participatory observation, multiple source data collection, and recursive interpretations [58, 64]. Each respondent personally answered the questions without any assistance, and there were no missing data. We acknowledge the potential bias in this study, in that we sampled attitudes after lion killing as opposed to before and these attitudes could have shifted with time. Nevertheless, they provided valuable insights into the probable triggers of lion killing.

### Analysis Variables

The scales explained above were based on a series of 22 statements (items) regarding general attitudes toward lions, traditional customs, and intentions toward lion killing (Table 1). The statements within each category were combined into a single additive score [62]. Answers to each statement were originally coded as follows: strongly disagree = -2, disagree = -1, unsure = 0, agree = 1, and strongly agree = 2. We summed the codes to create a combined score for each scale; for example, the higher the score in the attitude scale, the more positive attitudes were toward lions, whereas the higher the score in the killing intention scale, the higher propensity to kill a lion. The dependent variable used in the logistic regression was based on verified knowledge about the respondent’s lion killing history (killed = 0, not killed = 1).

**Table 1:** Items included in the factor analysis and the percentage of respondents who agreed/strongly agreed with each statement (n = 123).

**General attitudes toward lions**	**Agreement rate (%)**
a. I feel lions have the same rights as livestock to live on this land	55
b. I feel that lions are beautiful animals	47
c. God would want me to protect lions	52
d. Lions deserve protection	51
e. Lions have a right to exist	54
f. It is important to me that my grandchildren see lions	55
g. The lions in the ecosystem are a national treasure	50
h. I like to watch lions in their natural environment	48
**Attitudes toward traditional customs**	**Agreement rate (%)**
i. I will only eat livestock that has been suffocated	89
j. My daughters must be circumcised	80
k. I believe sacrifices to pray for rain are important	81
l. I do not own a bible	87
m. I do not wear western clothes	90
**Intentions toward lion killing**	**Agreement rate (%)**
n. If my cow was killed by a lion it is acceptable to kill it	45
o. Killing a lion for prestige/status is acceptable	22
p. When I see a lion it is acceptable to kill it	20
q. Snaring a problem lion is acceptable	48
r. Traditional hunts are acceptable	30
s. I will kill a lion just for fun	19
t. I will kill a lion to defend my property	86
u. If a lion entered by *boma (livestock corral)* I would kill it	75
v. If a family member was injured by a lion I would kill it	92

### Data Analysis

Exploratory Factor Analysis (EFA) with promax rotation was used to identify the number of factors (e.g., eigenvalues > 1). Following Tabachnick and Fidell [65], all loadings > .32 were retained and Cronbach’s alpha was used to determine the internal consistency of the scales, setting the criterion for good reliability at .70. Lastly, a binary logistic regression was used to determine the contribution of each factor in predicting actual lion killing. We tested the variance inflation factor and checked the variance decomposition proportions [66]; both tests confirmed that there was no collinearity present among the predictors and there was no evidence of outliers, influential observations, or heteroskedasticity.

## Results

### Respondent Characteristics

Respondents (*n* = 123) were all traditional warriors, ranging in age between 20–33 years. Ninety-three percent of the interviewees had never attended school, with the remaining 7% having completed primary school. The average residency of all respondents was 20 years in their communities. All respondents owned livestock, primarily for subsistence (89%), while 11% also owned them for sale. The average livestock holding was 12 cows, with a median of 7.5; this low number is common since most warriors do not own many livestock but care for their fathers herd. In addition, these respondents reported losing an average of 1.3 cows during a 12 month period to predators, with a median of 0 losses. For those respondents who had killed lions (*n* = 61), the total number of lions killed per respondent varied (mean of 2, range 1–12). Fifty-one percent stated cultural reasons (e.g., gaining prestige) as the main reason for killing a lion, while 49% said that they killed in response to actual livestock depredation.

### Factor Analysis

The EFA revealed four factors with eigenvalues exceeding 1. All items loaded significantly on only one factor (Table 2). The internal consistency of Factor 1 was high,.97, which indicated that the attitude items were measuring the intended construct. The traditional customs statements all loaded strongly onto Factor 2 with a Cronbach’s alpha of .85. The intentions toward lion killing scale split into two distinct factors (Factors 3 and 4; Table 2), indicating that Maasai perceptions of lion killing were distinguished by two types of intentions that do not overlap. One was a desire to reinforce the role of warriors in society, hereafter called ‘social-killing intention’ (Factor 3) while the other was defense of property and livestock, henceforth named ‘defense-killing intention’ (Factor 4). Both intention factors passed the Cronbach reliability test with strong alpha coefficients: .89 for social-killing intention and .85 for defense-killing intention.

**Table 2:** Pattern matrix of social predictors of lion killing: Four-factor model with promax rotation (n = 123).

Brief statements	Factors			
	1 Attitudes	2 Traditional customs	3 Social-killing intention	4 Defense-killing intention
Lions have a right to exist	.971	--	--	--
Lions deserve protection	.939	--	--	--
Important grandchildren see lions	.934	--	--	--
Lions are a national treasure	.935	--	--	--
God would want me to protect lions	.926	--	--	--
Lions have the same rights as livestock	.872	--	--	--
Lions are beautiful	.851	--	--	--
Like to watch lions	.833	--	--	--
Only eat suffocated livestock	--	.838	--	--
Do not wear western clothes	--	.835	--	--
Do not own a bible	--	.833	--	--
My daughter must be circumcised	--	.703	--	--
Believe in sacrifices for rain	--	.695	--	--
Kill lion for fun	--	--	.992	--
Kill lion for prestige	--	--	.910	--
Traditional hunts are acceptable	--	--	.882	--
When I see a lion it is acceptable to kill	--	--	.755	--
Kill lion to defend property	--	--	--	.898
Family member injured by lion	--	--	--	.880
Lion enter my boma	--	--	--	.765
Snaring a problem lion	--	--	--	.625

### Logistic Regression

To examine the combined influence of the variables on lion killing behavior, we used logistic regression (Table 3). The Nagelkerke *R*^2^ indicated that 84% of the variation in actual lion killing was explained, demonstrating the robustness of the model in predicting actual killing. Seventy-three percent of the variation in actual lion killing was explained by attitudes toward lions, while only 30% was explained by traditional customs, and both predictors were significant in the model (≤.001 and .006 respectively). Therefore, the more positive the respondent’s attitudes were toward lions, the less likely they were to engage in lion killing. The odds ratio (Exp(B), Table 3), suggests that there was a 38% increase in the odds that a non-killer had a higher (more positive) attitude score than a respondent who killed a lion. The two killing intention factors were not significant and explained less than 10% of the variation in actual lion killing behavior.

**Table 3:** Logistic regression model of variables predicting actual lion killing behavior (Negelkerke R^2^ = .84) (Dependent variable: killed = 0, not killed = 1).

*Predictors*	*B*	*S*.*E*.	*Wald*	*df*	*Sig*.	*Exp(B)*
Attitudes	.324	.072	20.185	1	≤.001	1.383
Traditional customs	-1.154	.420	7.546	1	.006	.316
Defense-killing intention	-.095	.094	2.073	1	.387	1.026
Social-killing intention	.174	.116	2.257	1	.133	1.190
Constant	5.060	1.865	7.362	1	.007	157.617

## Discussion

Humans have caused recent extirpations of many carnivore populations worldwide [5, 67], yet research devoted to carnivore conservation has tended to focus more on managing carnivore populations [68–70] than on influencing human behavior. However, there is an increasing recognition that solutions focused on carnivore biology alone limit conservationists’ ability to reduce human-induced killing, implying a need to focus management solutions on humans as well [71, 72].

Our findings suggested that Maasai attitudes toward lions, followed by traditional customs, best predict actual killing. This is not surprising, since attitudinal theory holds that cognitive processes tend toward consistency and that attitudes are reliable predictors of behavior [73–75]. However, there has been little previous empirical evidence for this link regarding lion killing. Similar results were found when documenting Maasai attitudes toward elephants in the Amboseli region [76]. This study indicated that attitudes may predict behavior in situations where local communities have strong knowledge about the species of interest, similar to findings from other studies [77, 78]. This is consistent with the Maasai context since warriors have an intimate knowledge and understanding of lions, both as a conflict species and one that brings them honor and prestige [56].

However, attitudes do not always predict positive conservation behavior. For example, Infield and Namara [79] found that although communities living around Lake Mburu National Park in Uganda had experienced long-term conservation benefits and expressed positive attitudes toward wildlife and conservation, they still continued to poach wildlife regularly. Similarly, Liu et al. [80] concluded that human-wildlife conflicts in China shaped people’s attitudes toward bears, but strong economic incentives (illegal trade in bear parts), not attitudes, prompted illegal killing. These studies all suggested that illegal killing of wildlife for either political-economic benefit or subsistence reasons may not be easily predicted by attitudes. However, studies have also suggested that retaliatory killing of carnivores in response to livestock depredation can be predicted by attitudes ([81], see [82, 83, 84]).

Our results suggested that general attitudes toward lions appear more important than intentions toward lion killing, e.g., in defense of livestock. This is an important result—conflict mitigation efforts often focus on reducing the costs imposed by carnivores through depredation [85, 86], but this study highlights a very close association between attitudes and lion killing. Therefore, improving local attitudes, in conjunction with appropriate mitigation techniques, may reduce lion killing most effectively. For example, Lion Guardians, a conservation organization based in Kenya, has documented a 99% reduction in lion killing by improving Maasai warrior attitudes toward lions through relying on strong cultural values, using traditional mitigation techniques to reduce conflict, and empowering warriors to participate in lion monitoring and conservation [57, 87].

## Conclusions

By expanding our understanding and influences on human behavior, we can improve conservation of threatened wildlife populations [72]. Our results suggested that attitudinal research that informs practice can enhance our ability to affect behavior toward wildlife, particularly if attitudes are monitored over time. Cultural and environmental variables specific to each local situation may drive and predict human behavior, underscoring the need for local data rather than dependence on literature based on studies elsewhere. In addition, many of the social-psychology tools have been developed in North America [31, 88, 89] and, because they focus on recreational sport hunting, may not be easily applied or contextualized in a rural African subsistence setting.

Our findings have implications for initiatives that attempt to alter human behavior towards carnivores, bolstering the common assumption that conservationists should invest resources and effort in improving local attitudes toward carnivores through both economic and non-economic benefits, including ownership of wildlife [52, 90–93]. Attitudes toward lions and lion killing behavior have been shown to improve with livestock compensation payments, employment and participation in conservation activities [93]. A recent study in the Masai Mara ecosystem in Kenya suggests that community conservancies that provide direct financial benefits to individual Maasai via tourism has resulted in an increase in lion numbers [24]. Similarly, western ranchers in Kenya embraced lion-friendly livestock management when they added wildlife tourism to their traditional ranching economy [19]. Furthermore, implementing proactive, culturally-appropriate mitigation techniques may help improve local tolerance towards wildlife, as will understanding the political, historical, and social drivers of conflict [94, 95]. Mechanisms will vary with local circumstances, but improving attitudes is an important conservation goal.

When trying to predict future behavioral outcomes, collecting information on why, when, and by whom specific behaviors, e.g. hunting/killing, are performed prove very beneficial when developing solutions for reducing illegal behavior [96]. This approach may include documenting narratives of real hunts, as these can reveal patterns of motivations and underlying cultural drivers of behavior [97]. This kind of research on human dimensions has great potential for improving practical interventions in conservation, as it highlights the key factors determining actual actions, and therefore allows the most appropriate strategies to be implemented to effectively reduce wildlife killing.

## Supporting Information

S1 Data Interview data analyzed for this study. (XLS)
